# Supplementary figures and images for: An iron-dependent metabolic vulnerability underlies VPS34-dependence in RKO cancer cells
Source: PLoS One. 2020 Aug 24;15(8):e0235551. doi: 10.1371/journal.pone.0235551 (PMC7446895; doi:10.1371/journal.pone.0235551)

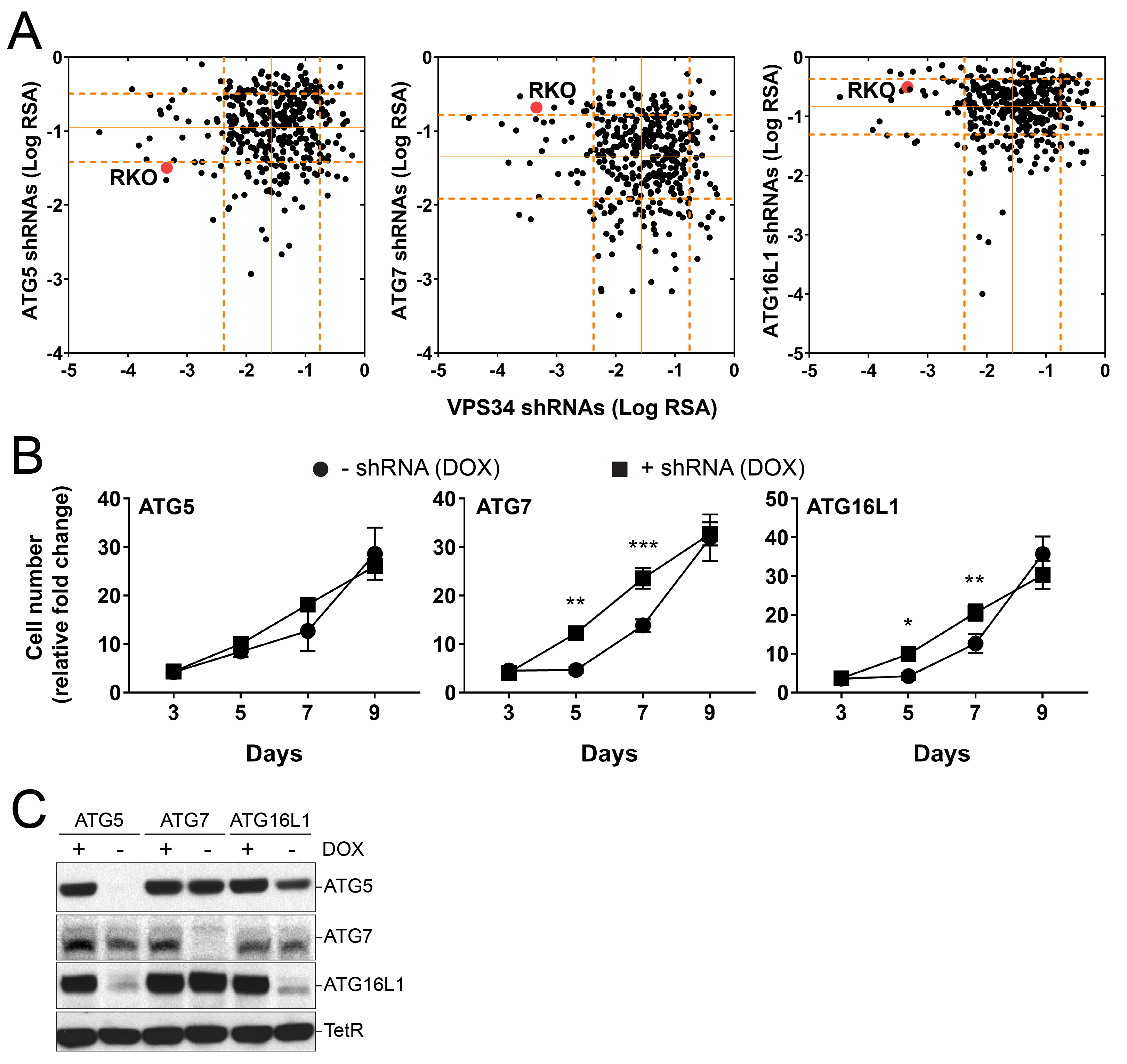

Supplement: S1 Fig — (A) Pooled shRNA screening data for VPS34, ATG5, ATG7, and ATG16L1 was extracted from project DRIVE [25] and visualized as RSA significance scores. Each dot represents a cell line and RKO is highlighted in red. The straight and dotted lines indicate average and 2x standard deviation of the RSA values across all cell lines, respectively. (B) Knockdown of canonical autophagy components (ATG5, ATG7 and ATG16L1) were performed in RKO cells expressing doxycycline (Dox) inducible shRNA targeting ATG5, ATG7 or ATG16L1. Cell proliferation was monitored by CellTiter-Glo assay as the mean of three independent experiments with error bars representing SD. *, p < 0.05; **, p < 0.01; ***, p < 0.001 (two-way ANOVA). (C) Characterization of RKO ATG5, ATG7 and ATG16L1 doxycycline (Dox) inducible shRNA cell lines. Cell lysates of RKO ATG5, ATG7 and ATG16L1 cells were immunoblotted with the specified antibodies. (TIF) [file pone.0235551.s001.tif]

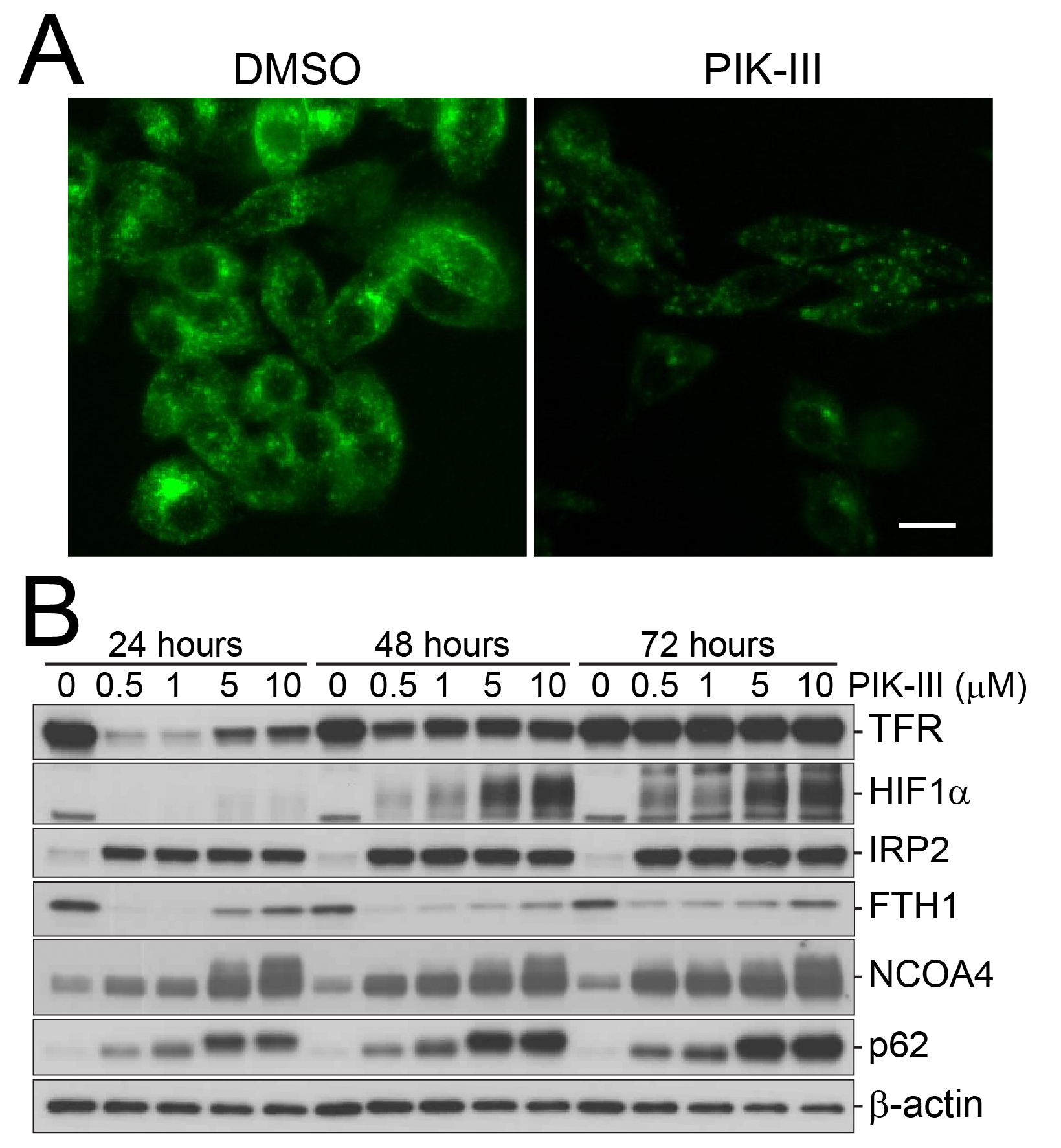

Supplement: S2 Fig — (A) RKO CTRL cells were treated with vehicle or 1 μM PIK-III for 72 hours followed by incubation with Transferrin Alexa Fluor 488 probe for 20 min at 37°C and live cell imaging was performed. The white bar represents 10 μm in length. (B) RKO CTRL cells were treated with the indicated concentrations of PIK-III for 12, 24 and 72 hours and lysates were immunoblotted with the indicated antibodies. (TIF) [file pone.0235551.s002.tif]

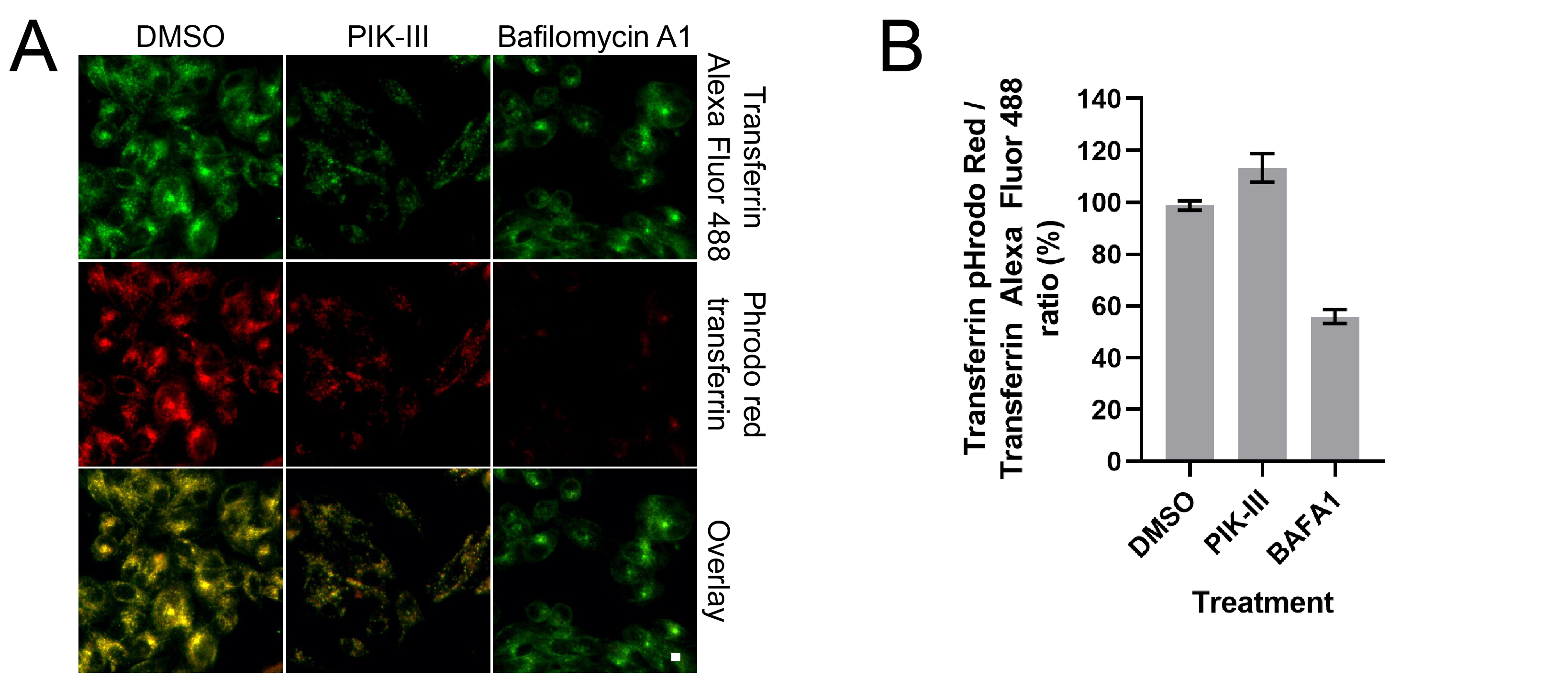

Supplement: S3 Fig — (A) RKO CTRL cells were treated with vehicle or 1 μM PIK-III for 24 hours and with 100 nM bafilomycin (BAFA1) for 4 hours. The cells were incubated with 1:1 mixture of the Alexa Fluor 488 and pHrodo Red transferrin probes for 20 min at 37°C before live cell imaging. The white bar represents 10 μm in length. (B) Images collected in (A) were quantified as the percent ratio of median intensity of pHrodo Red and Alexa Fluor 488 transferrin probes. Data was averaged and presented as the mean ± SD from 2 wells. (TIF) [file pone.0235551.s003.tif]

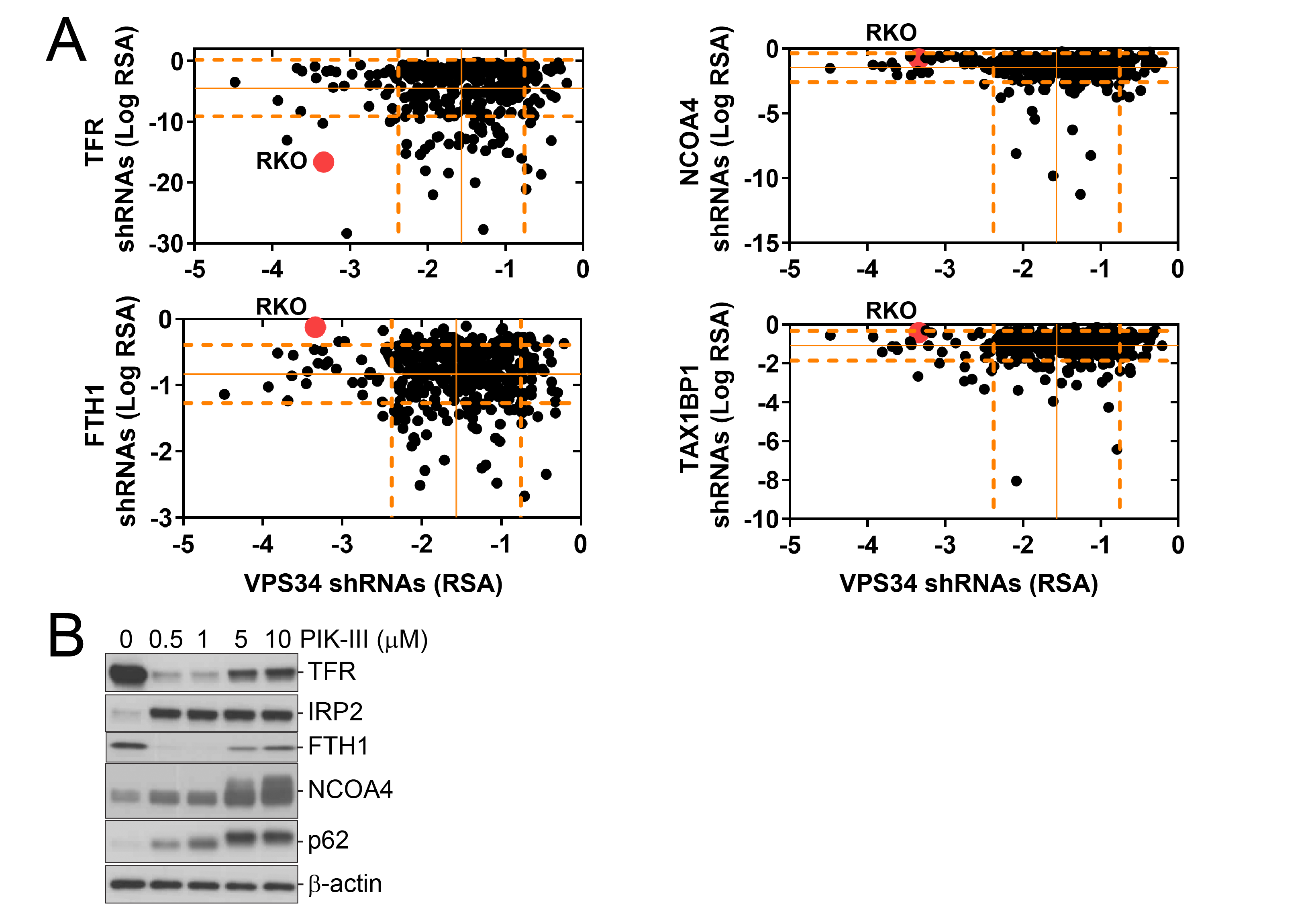

Supplement: S4 Fig — (A) Pooled shRNA screening data for VPS34, TFR, FTH1, NCOA4 and TAX1BP1 were extracted from project DRIVE [25] and visualized as RSA significance scores. Each dot represents a cell line and RKO is highlighted in red. The straight and dotted lines indicate average and 2x standard deviation of the RSA values across all cell lines. (B) Cell lysates of RKO CTRL cells treated with the indicated concentrations of PIK-III for 24 hours were immunoblotted with the specified antibodies. (TIF) [file pone.0235551.s004.tif]

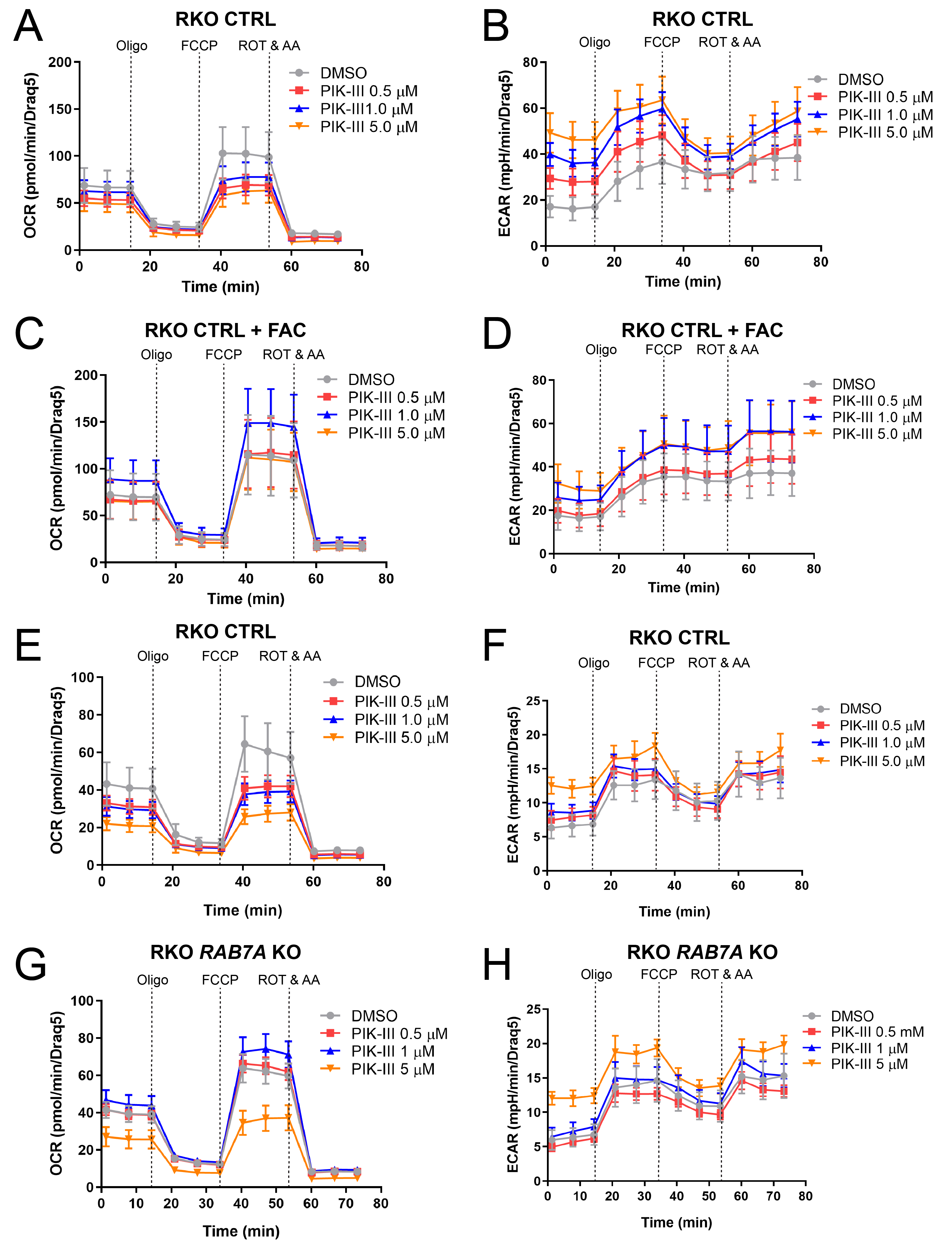

Supplement: S5 Fig — (A–D) Mitochondrial respiration defect due to PIK-III treatment. RKO CTRL cells were treated with the indicated concentrations of PIK-III along with vehicle for 24 hours and mitochondrial respiration rates for OCR, (A) and ECAR, (B) were assessed using the Seahorse XFe96 analyzer. Data in the form of technical replicates were averaged and presented as the mean ± SD (n = 11 or 12 wells). RKO CTRL cells were treated with the indicated concentrations of PIK-III along with 50 μM FAC for 24 hours and mitochondrial respiration was assessed by measuring the OCR, (C) and the ECAR, (D). Data in the form of technical replicates were averaged and presented as the mean ± SD (n = 11 or 12 wells). (E–H) Mitochondrial respiration defect due to VPS34 inhibition is RAB7A-dependent. RKO CTRL or RAB7A KO cells were treated with the indicated concentrations of PIK-III for 24 hours and mitochondrial respiration was assessed by measuring the OCR (E, G) or the ECAR (F, H). Data in the form of technical replicates were averaged and presented as the mean ± SD (n = 7 or 8 wells). (TIF) [file pone.0235551.s005.tif]

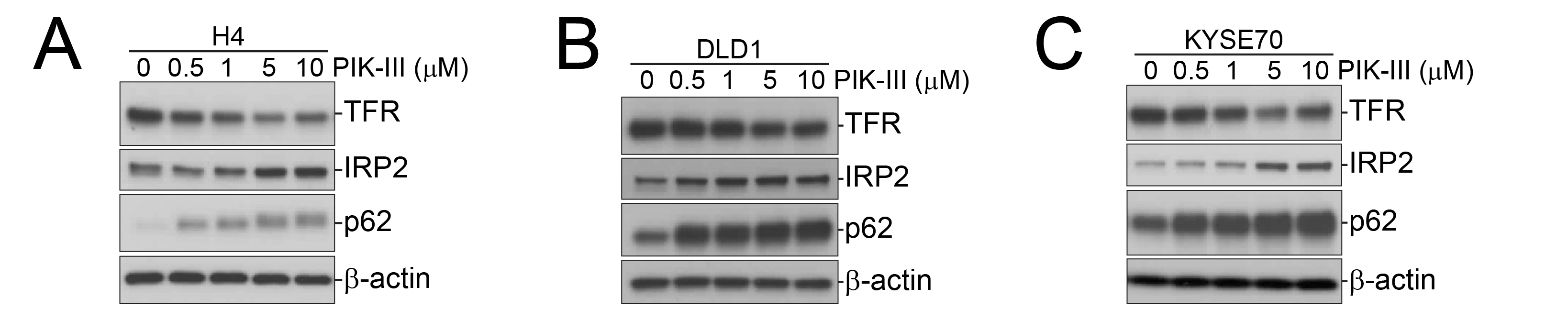

Supplement: S6 Fig — H4, DLD1 and KYSE70 cells were treated with the indicated concentrations of PIK-III for 24 hours and lysates were immunoblotted with the indicated antibodies. (TIF) [file pone.0235551.s006.tif]

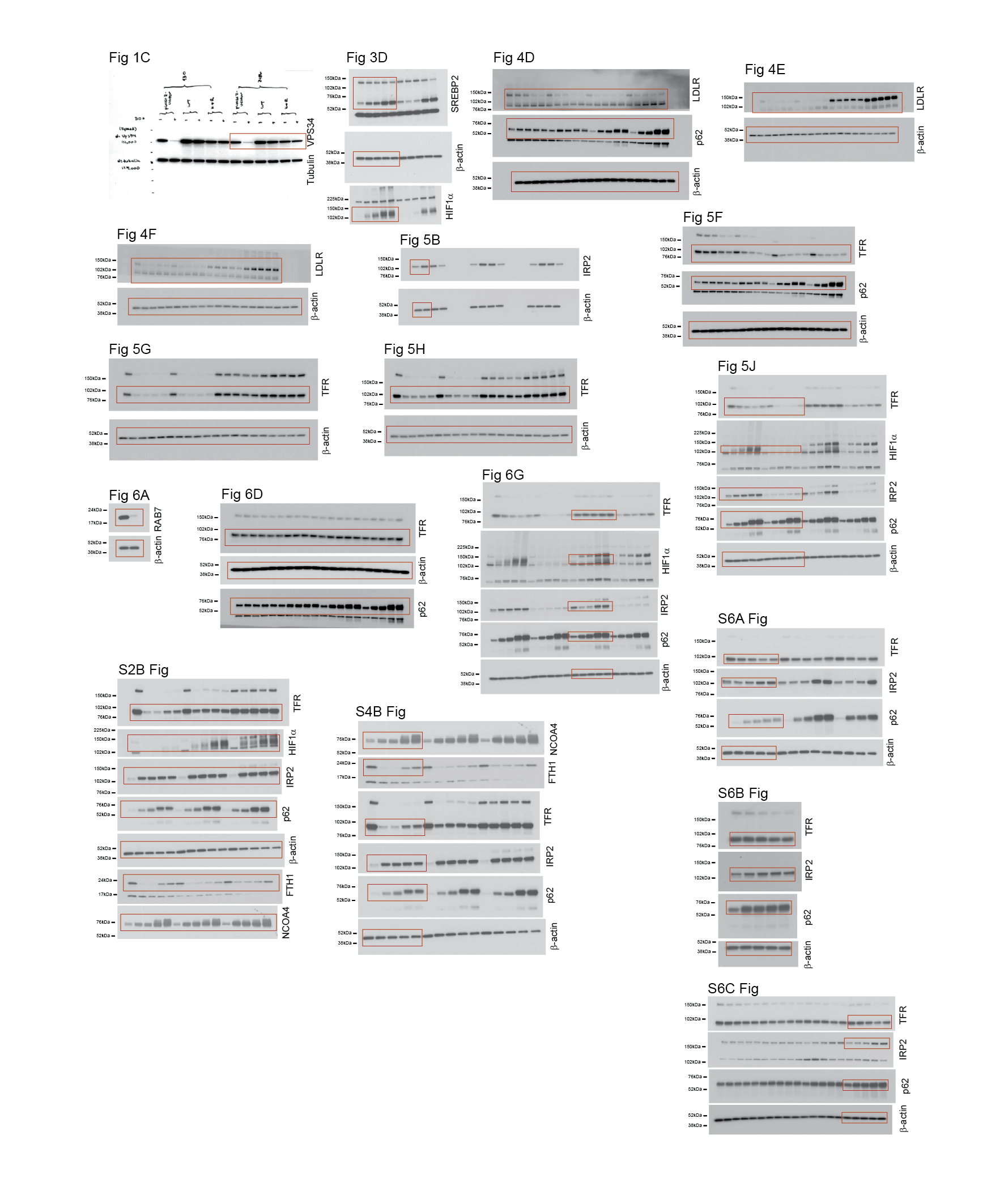

Supplement: S1 Raw images — (TIF) [file pone.0235551.s011.tif]
